# Hyperkalemic cardiac arrest induced by mannitol administration during craniotomy: A case report and review of the literature

**DOI:** 10.3389/fsurg.2022.1019101

**Published:** 2022-10-11

**Authors:** Hua Zheng, Xueqin Cao, Feng Gao, Xinhua Li, Li Wan, Ailin Luo

**Affiliations:** Department of Anesthesiology and Pain Medicine, Tongji Hospital, Tongji Medical College, Huazhong University of Science and Technology, Wuhan, China

**Keywords:** mannitol, hyperkalemia, cardiac arrest, craniotomy, complication

## Abstract

**Introduction:**

Mannitol is the most widely used hyperosmolar agent during neurosurgical procedures. However, its use can lead to serious hyperkalemia with altered cardiac conduction.

**Case presentation:**

Here we report a case in which a 40-min cardiac arrest was caused by mannitol-induced hyperkalemia during craniotomy. In addition, we conducted a literature review through a PubMed (MEDLINE) search of the relevant literature published so far. Details of all cases are presented and discussed. The results suggest that male patients or patients with uncontrolled diabetes might be at higher risk to develop this phenomenon. The results also suggest that the high dose and rapid rate of infusion of mannitol might contribute to mannitol-induced hyperkalemia.

**Conclusion:**

Physicians should be aware of the existence of mannitol-induced hyperkalemia. Although the mechanism of this complication is not well established, it is prudent to administer mannitol cautiously, especially in patients with uncontrolled diabetes. Continuous electrocardiogram monitoring and frequent measurements of serum electrolytes can help to detect and treat possible life-threatening events induced by mannitol-induced hyperkalemia early.

## Introduction

Mannitol, a sugar alcohol, is used commonly to reduce intracranial pressure and brain bulk in neurosurgical patients (1). Mannitol acts as an osmotic diuretic, increasing blood osmolality acutely, and shifting water from the intracellular and interstitial compartments into the intravascular space, thus reducing brain water content (2). This response decreases brain bulk and thereby the quality of surgical exposure (3). However, mannitol administration can lead to serious electrolyte abnormalities, especially hyperkalemia (4). Here we report a case of life-threatening hyperkalemia induced by mannitol administration during supratentorial brain tumor resection. In addition, we review the pertinent literature and discuss the prevalence, mechanism, risk factors, diagnosis, and treatment of mannitol-induced hyperkalemia.

## Case presentation

A 66-year-old (height: 175 cm, body weight: 72 kg) man presented with left leg weakness for 3 months and dizziness for 1 month. Brain magnetic resonance imaging showed a sphenoid wing meningioma with diffuse brain edema of the right hemisphere and subfalcine hernia. He had a history of type 2 diabetes that was managed using insulin. He smoked half to one pack of cigarettes daily for 45 years and had recently stopped smoking. Previous surgical procedures included lumbar discectomy, phacoemulsification cataract surgery, and partial prostatectomy. His physical examination was notable for left leg weakness. His lungs were clear to auscultation bilaterally on pulmonary examination. He had a normal heart rate and a regular heart rhythm on cardiac examination. Preoperative electrocardiogram and echocardiography were both normal. Preoperative laboratory tests showed a normal complete blood count and comprehensive metabolic panel except for high blood sugar.

**Figure 1:**
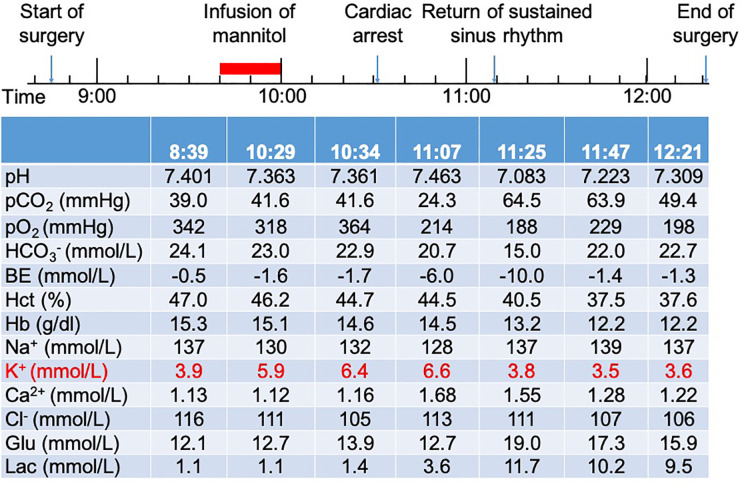
Timeline of surgery and results of arterial blood gas analysis of the patient.

The patient was scheduled for a brain tumor resection through the transtemporal approach. When he arrived in the operating room, his initial electrocardiogram showed normal sinus rhythm at 72 beats per minute (bpm) and his blood pressure was 140/88 mmHg. Other vital signs included: respiratory rate, 15 breaths per minute; oxygen saturation using pulse oximetry (SpO_2_) in room air, 98%; temperature, 37 °C. After preoxygenation and anesthetic induction with sufentanil, cisatracurium, and propofol, endotracheal intubation proceeded uneventfully. Then a right femoral venous catheter was placed, as well as a left dorsalis pedis arterial catheter. Arterial blood gas and electrolyte analysis showed a pH of 7.401, a partial pressure of carbon dioxide (PaCO_2_) of 39 mmHg, a partial pressure of oxygen (PaO_2_) of 342 mmHg, a sodium concentration of 137 mmol/L, and a potassium concentration of 3.9 mmol/L (Figure 1).

Anesthesia was maintained using 1.5%–2% sevoflurane with 60% O_2_ and intraoperative analgesia was achieved with an infusion of remifentanil. The patient was hemodynamically stable at the beginning of surgery. After the skin incision, 250 ml of 20% mannitol (0.7 g/kg) was administered intravenously over a period of 20 min. Approximately 30 min after completion of the mannitol infusion, the patient's blood pressure decreased from 130/80 to 90/60 mmHg and electrocardiograph showed peaked T waves. An arterial blood sample was drawn and showed a pH of 7.363, a partial pressure of carbon dioxide (PaCO_2_) of 41.6 mmHg, a partial pressure of oxygen (PaO_2_) of 318 mmHg, a sodium concentration of 130 mmol/L, and a potassium concentration of 5.9 mmol/L. A repeat sample for arterial blood gas and electrolyte analysis was sent and the potassium concentration was 6.4 mmol/L. The patient rapidly developed ventricular fibrillation followed by asystole. Cardiopulmonary resuscitation (CPR) with chest compressions was initiated immediately. First, 1 mg of epinephrine, 1 g of calcium gluconate, and 10 U of regular insulin were given intravenously. Then 250 ml of 5% sodium bicarbonate was infused. Repetitive attempts at electrical defibrillation were made and the patient intermittently regained normal sinus rhythm. After 40 min of CPR, the patient returned to sustained sinus rhythm while his potassium decreased to 3.8 mmol/L.

After surgery, the patient was conveyed to an intensive care unit. The postoperative evaluation did not show pulmonary embolism, myocardial infarction, or any other cardiovascular abnormalities. The patient was tracheally extubated on postoperative day 1 and transferred to a general ward on postoperative day 3. Two weeks later, the patient was discharged home without neurologic deficits or clinical sequelae.

## Literature review

A literature review was conducted through a PubMed (MEDLINE) search of the literature published up to August 1, 2022. “Hyperkalemia” AND “mannitol” were used as search terms. No language and start date restrictions were applied. Further studies were identified by screening the references of publications that covered the topics. Those studies that did not accurately describe the clinical presentation, diagnostic process, and therapeutic procedures were excluded. A total of 11 relevant publications with 14 case reports were included (Table 1).

**Table 1:** Details of all cases of mannitol-induced hyperkalemia.

Reference	Characteristics of patients			Infusion of mannitol		Electrocardiogram changes		Potassium concentration (mmol/L)			Treatment
	Age (years)	Sex	Diagnosis	Dose g (g/kg)	Duration (minutes)	Onset time^a^ (minutes)	Manifestations	Baseline	Peak	End of surgery	
1 (5)	52	M	Artery aneurysm	60 (1.0)	20	60	Peaked T waves, widened QRS, VT	4.8	6.8	5.0	Insulin with glucose, lidocaine, calcium
2 (6)											
Patient 1	34	M	Artery aneurysm	60 (1.0)	20	30	Peaked T waves	3.1	5.4	3.5	Hyperventilation, calcium, insulin with glucose
Patient 2	68	M	Intracerebral hemorrhage	100 (1.3)	45	90	Peaked T waves, bigeminy	4.1	6.1	5.4	Lidocaine, bicarbonate, hyperventilation
3 (7)	15	M	Arteriovenous malformation	60 (1.1)	NA	45	Peaked T waves, widened QRS	5.3	6.7	5.2	Calcium, bicarbonate
4 (8)	31	F	Astrocytoma	80 (1.0)	45	60	Peaked T waves	4.1	6.1	4.5	Calcium, insulin with dextrose
5 (9)	41	M	Cerebral aneurysm	120 (NA)	40	40	Peaked T waves, widened QRS, VT, VF, asystole	NA	7.5	3.9	Calcium, bicarbonate, insulin with dextrose
6 (10)	43	M	Cerebellar tumor	60 (NA)	NA	30	NA	4.8	6.7	4.0	NA
7 (11)	23	M	Cerebellar hemangioblastoma	NA (1.5)	20	30	Peaked T waves, widened QRS	NA	9.0	4.1	Calcium, insulin with dextrose, furosemide
8 (12)											
Patient 1	57	M	Artery aneurysm	45 (0.7)	15	125	Peaked T waves	4.0	6.0	4.4	Furosemide, bicarbonate
Patient 2	49	M	Artery aneurysm	30 (NA)	20	170	Peaked T waves	3.7	5.7	5.0	Furosemide, bicarbonate
9 (13)	43	M	Metastatic tumor	80 (0.7)	20	80	Peaked T waves, widened QRS, VT, VF, asystole	4.2	6.5	6.1	None
10 (14)	62	M	Artery aneurysm	40 (0.6)	60	80	VT, peaked T waves	4.1	6.4	3.5	Calcium, furosemide, insulin with glucose
11 (15)											
Patient 1	69	M	Cerebellar tumor	50 (0.8)	NA	25^b^	VF, asystole	4.2	6.0	5.8	Bicarbonate
Patient 2	69	M	Artery aneurysm	50 (0.6)	NA	None	None	4.4	6.4	5.1	Insulin
Current report	66	M	Sphenoid wing meningioma	50 (0.7)	20	50	Peaked T waves, VF, asystole	3.9	6.6	3.6	Calcium, insulin, bicarbonate

^a^ Time between the start of mannitol infusion and onset of electrocardiogram changes.

^b^ Time between completion of mannitol infusion and onset of electrocardiogram changes.

F, female; M, male; NA, not available; VF, ventricular fibrillation; VT, ventricular tachycardia.

## Discussion

Mannitol has been used to reduce intracranial pressure and volume for 60 years. However, the use of mannitol can cause life-threatening adverse effects. Here, we report a case in which hyperkalemic cardiac arrest caused by infusion of mannitol in a patient with uncontrolled diabetes. Clinical features and treatments of this case are described and discussed in the context of the relevant literature. This case report and literature review indicates the importance of early recognition and rapid correction of mannitol-induced hyperkalemia.

The occurrence of significant hyperkalemia after administration of mannitol was relatively rare but life-threatening. Most of the cases reported in the literature were from Asia, especially Japan. According to preoperative diagnosis, nine (60%) of the patients had cerebrovascular diseases, and six (40%) had cerebral tumors. The ages of the patients ranged from 15 to 69 years, and the mean age was 48 years. Of the 15 patients, 14 (93%) were male and only 1 (7%) was female. These results suggest that male patients might be at higher risk of lethal mannitol-induced hyperkalemia.

The mechanism of the rise in serum potassium following mannitol is incompletely identified. Some possible mechanisms have been offered to explain this phenomenon. First, hemolysis due to the direct effect of hypertonic mannitol on the red blood cells. However, Evers et al. showed that the survival time of the red blood cells was not adversely affected after the injection of mannitol (16). The results from all published case reports and us also did not suggest hemolysis to account for the increase in potassium. Second, acidosis secondary to dilution of bicarbonate, which is attributed to a temporary intravascular volume expansion after mannitol administration. Third, physiological response to a rise of H^+^ concentration. According to the strong ion difference theory, hypertonic mannitol decreases sodium reabsorption and the strong ion difference with a resultant increase in H_2_O dissociation and H^+^ concentration (17). However, in a retrospective study by Hirota et al., no correlation between mannitol-induced hyperkalemia and acid-base abnormalities was detected (6). Previous case reports and ours also did not support the second and third proposed mechanisms.

Another mechanism of mannitol-induced hyperkalemia that has been considered is a solvent drag. According to this theory, increased intracellular osmolality and intracellular water loss result in the movement of water out of cells and the shift of potassium along with it. In the current report, impaired function of glucoregulatory hormones may also play an important role in mannitol-induced hyperkalemia. Under normal circumstances, increased serum potassium stimulates insulin release which then puts potassium into the cells (18). But this response is impaired in uncontrolled diabetes mellitus. In addition, marked hyperglycemia also accounts for hyperkalemia. Thus, in patients with uncontrolled diabetes mellitus, abandonment of or caution with the use of mannitol is recommended.

It is reported that mannitol-induced hyperkalemia is dose-dependent (19). The doses of mannitol used in the current literature review vary from 0.6 to 1.5 g/kg. However, a systematic study indicated that an equivalent intracranial pressure reduction can be achieved with 0.25 g/kg of mannitol while avoiding the risk of severe adverse effects (20). In addition, the rapid rate of infusion of mannitol may also contribute to mannitol-induced hyperkalemia. Most patients presented in Table 1 received mannitol within 20 min, even though a high dose of up to 80 g of mannitol was administered. The dose and rate of infusion of mannitol as risk factors were also confirmed in the current report, as the patient had received preoperatively 0.3 g/kg mannitol over 30 min without any adverse effect in general ward. Collectively, high doses and rapid rates of infusion of mannitol should be avoided during craniotomy. In patients with contraindications of mannitol, hypertonic saline might be used as an alternative (21).

The changes in electrocardiogram, such as peaked T waves and widened QRS, were usually the first finding of mannitol-induced hyperkalemia. However, these changes did not occur in every case and developed quickly to lethal arrhythmia. In addition, the time between the start of mannitol infusion and the onset of electrocardiogram changes ranged from 30 to 170 min. Thus, mannitol should be administered with continuous electrocardiogram monitoring and repeated arterial blood gas analysis throughout the surgical procedures. When mannitol-induced hyperkalemia occurs, it is a medical emergency that requires rapid treatment. Based on the onset time and mechanisms, Calcium, insulin, and bicarbonate were the first choices to treat mannitol-induced hyperkalemia.

## Conclusion

Hyperkalemia is a rare but life-threatening complication of mannitol administration during craniotomy. This phenomenon is more likely to occur in male patients. Although its underline mechanisms remain unknown, high doses and rapid rates of infusion of mannitol may serve as risk factors. Continuous electrocardiogram monitoring and repeated arterial blood gas analysis are essential after mannitol administration. When mannitol-induced hyperkalemia occurs, early recognition and rapid correction of hyperkalemia may prevent harmful events.

## Data Availability

The original contributions presented in the study are included in the article/Supplementary Material, further inquiries can be directed to the corresponding author.
